# Rational Emotive Behavior Therapy for exercise: examining self-determined motivation, alongside readiness, confidence, and motivation to exercise

**DOI:** 10.3389/fpsyg.2025.1557885

**Published:** 2025-07-15

**Authors:** Martin J. Turner, Nick Frost, Leon Outar, Helen O'Connor, Renátó Tóth, László Tóth, Nanaki Chadha, Andrew G. Wood

**Affiliations:** ^1^School of Psychology, Manchester Metropolitan University, Manchester, United Kingdom; ^2^Private Consultant, London, United Kingdom; ^3^School of Doctoral Studies, Hungarian University of Sport Science, Budapest, Hungary; ^4^Teacher Training Institute, Hungarian University of Sport Science, Budapest, Hungary; ^5^Department of Psychology and Sport Psychology, Hungarian University of Sport Science, Budapest, Hungary; ^6^Private Consultant, New Delhi, India

**Keywords:** applied psychology, behavior therapy, exercise, intervention, single-case study

## Abstract

**Introduction:**

The present paper aims to extend the scant research into REBT within an exercise population. A growing body of research supports the application of Rational Emotive Behaviour Therapy (REBT) theory and practice in exercise populations. However, very few research studies have tested the effects of REBT on exercise promotion. One vehicle through which REBT might facilitate exercise engagement is through mechanisms associated with self-determination theory (SDT).

**Methods:**

In the current paper, using a staggered multiple-baseline across-participants single-case experimental design, REBT is applied with two non-exercising participants via video conferencing. We wanted to test the effects of REBT on participants' irrational beliefs, self-determined motivation, readiness, confidence, and motivation for positive change in exercise behavior.

**Results:**

Visual and statistical analyses indicate that from pre to post REBT, participants reported reduced irrational beliefs, increased self-determined motivation, and increased readiness, confidence, and motivation for positive change. Findings are supported by social validation thematic analysis, in which positive changes in exercise behavior and motivation to exercise were revealed.

**Discussion:**

The novelty of this paper is in its application of REBT (a specific form of CBT) to exercise participants, a rarely studied population in REBT literature. It is hoped that the paper helps the field to take another step towards the applicability of REBT in exercisers by adding to only three previous empirical works. The robust design and triangulation of analysis offers considerable value to the existing body of research suggesting that REBT is a promising one-to-one strategy for promoting exercise initiation in sedentary populations.

## Introduction

Government health bodies continue to press the need for populations to increase exercise participation (Gov.UK., 2022; Fletcher et al., 2018). In a recent study sedentary behavior was found to be responsible for 70,000 deaths in the United Kingdom (U.K.) each year (Heron et al., 2019) and a report by the U.K. chief medical officers (Department of Health Social Care, 2019) indicates an increasing body of evidence that sedentary behavior and low exercise participation in adults is linked to an increase in life ending diseases and other chronic conditions such as obesity and diabetes II. The report also suggests that even small amounts of exercise can have a positive impact on health, stating “if physical activity were a drug, we would refer to it as a miracle cure” (p. 3).

The beneficial effects of exercise are supported by a wide range of evidence in many contexts. Kandola et al. (2019) found that regular exercise promoted an anti-depressant effect regulating pathophysiological conditions such as inflammation, oxidative stress, and neuroendocrine homeostasis. Mandolesi et al. (2018) found individuals that partake in regular physical exercise have improved cognitive functioning, enhanced mood, and improved wellbeing. Stubbs et al. (2017) reported that individuals who engaged in low physical activity were 32% more likely to experience debilitative anxiety than a high physical activity group. A meta-analytic review by Lee (2018) found regular exercise decreased the risk of dementia while inactivity was associated with an increased risk, these results are further supported by longitudinal research into the risk reducing effects of regular exercise on dementia (Zhou et al., 2017). Despite the benefits of regular exercise engagement, many adults still struggle to exercise regularly (Herning et al., 2005). There are several factors that can create obstacles to exercise such as low motivation, self-efficacy, competence, body dissatisfaction, poor mental health, and environmental qualities; such as access to facilities, time barriers, and social and cultural support (Dalle Grave et al., 2011). As such, much research has elucidated psychological determinants for physical activity including self-efficacy, social support, motivational styles, and perceived benefits (Dalle Grave et al., 2011). Research indicates that cognitive processes play a key role in the adoption and adherence to exercise (Prochaska and Marcus, 1994).

## Cognitive-behavioral approaches

Ways in which we can help individuals to healthily undertake exercise are of high value, for the individuals themselves, but also societally. Much research has investigated workplace interventions (Ginoux et al., 2019; Jakobsen et al., 2017), structured exercise programs (Cronholm et al., 2017), and the use of cognitive-behavioral approaches in more clinical settings. Cognitive behavioral therapy (CBT) (Ellis, 1957; Beck, 1995) is a well-established family of psychotherapies for which research indicates effectiveness in treating depression, anxiety disorders, and eating disorders (David et al., 2018). CBT principles place emphasis on identifying and managing maladaptive cognitive processes that hinder wellbeing and goal-attainment to bring about change. However, there are very few studies documenting the use of CBTs in improving exercise adherence and adoption, of the few, research has illustrated the CBTs can bring about improvements in exercise frequency and duration, adherence (Herning et al., 2005). CBTs employ a broad range of cognitive, emotive and behavior techniques to bring about change such as cognitive reconstruction, exposure, scheduling, problem solving, self-monitoring, which research suggest could be helpful in exercise adherence (Herning et al., 2005). One CBT for which there exists sparse research in the exercise literature is Rational Emotive Behavior Therapy (REBT) (Ellis, 1994).

Research into REBT theory and practice is growing, with positive effects being reported on exercise related outcomes, but no papers reporting the effects of REBT on exercise engagement. REBT enables participants to exercise control over their thoughts, emotions, and behaviors by adopting a rational philosophy toward life (Turner, 2022). This is largely achieved through the GABCDE framework, which emphasizes that the consequences (C) experienced by an individual (e.g., emotions) do not arise directly from adversity (A) itself. Instead, they are shaped by our beliefs (B) related to the activating event (A) and our underlying goals (G). In practice, the disputation (D) of irrational beliefs, followed by the development of rational, effective beliefs (E), can play a pivotal role in transforming exercise-related emotions and behaviors. The extant research indicates that REBT has been effective in reducing exercise dependence (Knapp et al., 2023; Outar et al., 2018) and irrational beliefs, which are the key cognitive component of REBT, and are related to greater exercise dependence (Miller et al., 2023). Away from exercise populations, REBT has been shown to be effective in producing positive health and wellbeing outcomes across non-clinical and clinical populations (see David et al., 2018; Jordana et al., 2023, for reviews). The positive effects of REBT are credited in part to its focus on weakening irrational beliefs, which underpin dysfunctional emotions and maladaptive behaviors (Szentagotai and Jones, 2010), and so weakening them appears to confer benefits upon those engaging in REBT.

## Self-determined motivation

There is still some debate about potential mechanisms of change when applying REBT and the extent to which irrational beliefs are proximal to wellbeing outcomes (Caserta et al., 2010; Turner, 2016). Contemporary research indicates that the relationship between irrational beliefs and wellbeing might act through some important mediators, such as automatic thoughts (Buschmann et al., 2018), thought suppression (Szentagotai, 2006), maladaptive schema (Turner et al., 2019), self-confidence (Mansell and Turner, 2022), and cognitive appraisals (Chadha et al., 2019, 2024). One such potential mechanism is self-determined motivation regulation (Turner, 2016), or the extent to which an individual's behavior is regulated through self-determined motivation motives. To explain, in a person-centered approach to cross-sectional data in a sample of exercisers, latent profile analysis (LPA) revealed two profiles, one characterized by high irrational beliefs and low self-determined motivation (high amotivation, high controlled motivation regulation, low self-determined motivation motivation regulation), and one characterized by low irrational beliefs and high self-determined motivation (low amotivation, low controlled motivation regulation, high self-determined motivation motivation regulation; Turner et al., 2022). Further, exercisers with a profile of high irrational beliefs and low self-determined motivation were more likely to report greater psychological distress. In a sample of ultra-marathon athletes, it was found that greater irrational beliefs were related to lower self-determined motivation (Miller et al., 2022). Further, irrational beliefs and self-determined motivation were related to sport anxiety, such that higher irrational beliefs and lower self-determined motivation were related to greater anxiety.

Aside from cross-sectional offerings, applied REBT studies have also examined self-determined motivation as an outcome of REBT. In brief, research conducted with athlete participants has shown that when REBT is applied to reduce irrational beliefs, there is also a reported increase in self-determined motivation. This effect has been demonstrated in triathlon (Davis and Turner, 2020; Turner and Davis, 2019), American football (Chrysidis et al., 2020), and archery (Wood et al., 2017). Finally, O'Connor (2018) presented an applied single-case study in which REBT reduced the irrational beliefs of a female experiencing exercise avoidance, and subsequently increased her regular exercise class attendance. In sum, it would appear that there is something about helping individuals to weaken their irrational beliefs that enables concurrent and or subsequent increases in self-determined motivation. Turner (2016, 2022) suggests that there is some conceptual overlap between irrational beliefs and the motivation regulation types captured in organismic integration theory (OIT), that might explain these applied findings.

In OIT, motivation is categorized across a continuum of five regulation types; intrinsic motivation, integrated regulation, identified regulation, introjected regulation, and external regulation. Also, individuals can lack intentionality and motivation toward an activity, reflected in amotivation (Gustafsson et al., 2018; Ryan and Deci, 2017). Intrinsic, integrated, and identified regulations are considered more self-determined, whilst introjected regulation and external regulation are considered less self-determined forms of motivation (Howard et al., 2020; Ryan and Deci, 2000). Amotivation is a lack of intention to enact a behavior (Ryan and Deci, 2000).

Research evidence indicates that more self-determined motivation regulation is related to greater psychological and physical health (Ng et al., 2012), sustained physical activity engagement and health markers (e.g., Emm-Collison et al., 2020). Research also suggests that greater autonomy promotes positive exercise behaviors and adherence (Wilson et al., 2008), and that interventions that increase self-determined motivation increase psychological health and health behaviors (Ntoumanis et al., 2020), and controlled motivation regulation is related to elevated burnout, and decreased engagement (De Francisco et al., 2020). In exercise populations, studies have found that more self-determined forms of behavioral regulation predict behavioral maintenance such as physical activity participation (Fortier et al., 2007; Frederick et al., 1996; Silva et al., 2011) and exercisers maintaining their exercise behavior report greater self-determined motivation to exercise than those in the preparation and action stages of exercise engagement (Thøgersen-Ntoumani and Ntoumanis, 2006). Daley and Duda (2006) found that individuals who were more self-determinably motivated reported increased early-stage exercise participation, and Mullan and Markland (1997) found more self-determinably regulated motivations were associated with later stage exercisers. A systematic review by Teixeira et al. (2012) indicates consistent support for the positive relationship between self-determined motivation and exercise. The authors also reported that identified regulation predicts short-term exercise adoption more strongly than intrinsic motivation, but that intrinsic motivation predicts long-term exercise adherence. In sum, greater self-determined motivation appears to be desirable for exercise uptake and maintenance.

## The present study

The chief purpose of this paper is to meet a research need in the REBT and exercise literature, by examining the effects of REBT on self-determined exercise motives, and subsequent readiness, confidence, and motivation to engage in exercise. Exercise behavior was of concern to the founder of REBT, Dr. Albert Ellis. Ellis rarely contributed to the sport and exercise psychology literature (see Ellis, 1982, 1994), but in his 1994 paper, Ellis advocates the use of REBT for exercise avoidance, suggesting individuals that hold irrational beliefs often seek to avoid uncomfortable situations. Irrational beliefs such as “I must be in control” or “others must not look down on me” may lead to ego or discomfort anxiety whereby individuals avoid exercise as they believe they will not be able to meet their demanded beliefs which would be awful, intolerable, and prove themselves to be a failure (Ellis and Dryden, 2007). Data to test Ellis' conjecture is thin, despite recent the aforementioned recent applied research (Knapp et al., 2023; Outar et al., 2018) and case study work demonstrating the potential utility of REBT for promoting exercise behavior (cf. O'Connor, 2018). Therefore, the present paper aims to extend the scant research into REBT within an exercise population.

Whilst there are examples of CBTs being used to promote exercise behavior (e.g., Herning et al., 2005; Schneider et al., 2004), REBT is a specific type of CBT, that is quite distinct from many other CBT-based frameworks (Turner et al., 2020a) because it focuses on a set of four specific irrational beliefs, a facet not share by other CBTs. Thus, the novelty of the proposed paper is that it applies this specific CBT (i.e., REBT) to exercise participants, a rarely studied population in REBT literature. It therefore helps us to take another step toward the applicability of REBT in exercisers by adding to only three previous empirical works (Knapp et al., 2023; Outar et al., 2018). In this paper, we focus on participant perceptions of exercise behavior, applying REBT to aid the movement from maladaptive perceptions of exercise (including irrational beliefs, motivation regulation, readiness, confidence, and motivation to engage in exercise). We aim to examine to extent to which applying REBT with non-exercise engaged participants can facilitate a positive change in exercise behavior. Based on previous literature (e.g., Lyon et al., 2012) it is hypothesized that REBT will facilitate a reduction in irrational beliefs and an increase in self-determined motivation (e.g., Davis and Turner, 2020), and subsequent increases in readiness, confidence, and motivation to engage in exercise.

## Methods

### Design

The study utilized an idiographic single-case, staggered multiple-baseline across participant A-B design (Barker et al., 2011) which has been used in previous REBT research to examine the effectiveness of REBT in exercise populations (Outar et al., 2018) and athletes (Turner and Barker, 2013). Methodologically, single-case designs are favorable because they allow the meticulous examination of each participant, and the data from a few subjects provides a comprehensive narrative (Normand, 2016). An idiographic design enables the reporting of intervention effects for each participant (Neil et al., 2013), and can link previous theory in an ecologically rich manner (Willig, 2013). It also enables specific measurement and assessment of target variables (Barlow and Nock, 2009), ideal for monitoring and evaluating interventions (Lyon et al., 2012). In a staggered multiple-baseline across participant A-B design, participants establish a stable baseline before prior to intervention. In the current study, this was done over a 4–5-week data collection period. This allows for meaningful conclusions and reliable statistical analysis in which baseline can be confidently compared against any changes due to the intervention (Barker et al., 2020; Hrycaiko and Martin, 1996). The A-B design is a robust procedure for assessing effect of the intervention (i.e., REBT) on the target variables, allowing researchers to ascertain whether and to what extent the intervention brought about change (Kazdin, 1982). The REBT intervention was applied in sequence across the two participants, meaning that participant 1 and 2 commenced REBT at different time points. This can allow for changes in the dependent variables to be more confidently attributed to the intervention rather than extraneous variables (Kazdin, 1982). Participant 1 started the REBT intervention in week 5 (after seven baseline data collection points over 4 weeks), which was 1 week ahead of participant 2, who started the REBT intervention in week 6 (after 10 data collection points over 5 weeks). Staggering the start allowed the researchers to rule out alternative explanations for the observed target variable changes and thus increasing internal validity (Barker et al., 2013). One would expect change to occur in the target participant(s) only, with the participant's data in the baseline phase remaining stable (Barker et al., 2011).

Early REBT research (Nelson, 1977; Goldfried and Sobocinski, 1975) discounted the value of single-case studies instead favoring large “n” experiment designs that lacked validity. But single-case study designs compliment the eclectic idiographic nature of human beings and their interaction with psychological interventions. Single-case studies also promote relevancy, meaning and replication which is important when utilizing findings into practical settings (Gorczynski, 2013). Done properly, single-case designs allow the repeated measurement of psychological phenomena, lending confidence to observed effects. For example, in Turner and Barker (2013), across four participants, 6, 7, 8, and 10 baseline datapoints (respectively) are taken prior to intervention, rather than just one as is typical in group-based designs. By increasing rigor and validity through well-designed and executed studies there are real world benefits for using idiographic single-case studies (Barker et al., 2011, 2020). Further, some scholars (e.g., Normand, 2016) believe that an overreliance on group comparisons is significant problem in psychology, and that we can learn more from studying fewer participants. Indeed, in REBT research that has taken a single-case design approach, detailed studies involving as little as three participants (Chrysidis et al., 2020; Cunningham and Turner, 2016), two participants (Maxwell-Keys et al., 2022; Turner, 2022), and one participant (Wood et al., 2017, 2020). In addition, whilst single-case designs are often championed for their internal validity, critics suggest that they lack external validity. However, as Thorngate (1986) points out, “To find out what people do in general, we must first discover what each person does in particular, then determine what, if anything, these particulars have in common…” (p. 75). Rather than leap ahead with between-groups designs under the guise of external validity, there is much to be gained from examining the particulars of how individuals engage with and react to interventions. Then, once we have discovered what works, why, how, and for whom, larger-scale studies can be employed to examine generalizability across a population.

Data were collected over a 4-month period across three data collection phases: baseline, intervention, and post-intervention. Participants completed self-report measures iPBI and the Behavioral Regulation in Exercise Questionnaire 3 (BREQ3; Markland and Tobin, 2004) twice weekly during the baseline phase, and once weekly during the intervention phase (3–4 days after each session), and post-intervention phase. Participants completed the readiness, confidence, and motivation to change questionnaires, and regular exercise questionnaire at intervention and post-intervention phases. All questionnaires were completed online using Qualtrics experience management software.

### Participants and selection criteria

Following university ethical approval, prospective participants were recruited through social media and a poster displayed at a local (U.K.) General Practitioner (physician) surgery. A total of 8 participants responded and took part in the screening process as has been adopted in previous similar research (e.g., Davis and Turner, 2020). All eight participants completed informed consent forms and were emailed information about the study prior to completing a measure of irrational beliefs (irrational performance beliefs inventory; IPBI, Turner et al., 2018) and indicated their future exercise intentions. In line with recommendations based on norm levels of iPBI-measured irrational beliefs, scores above 18 out of 35) were considered sufficient to warrant REBT intervention. From the eight participants screened, the two with the highest iPBI scores were selected for the REBT intervention. Only two were selected in order to facilitate the idiographic and detailed analysis of intervention effects whereby more could be learned by studying fewer participants (Normand, 2016).

The mean iPBI composite score across the screening population (*n* = 8) was 25.3. Participant 1 was a female aged 38, living in Scotland, married with two children, and scored a composite iPBI score of 28.25. Participant two was a male aged 34, living in England, with a partner and no children, and scored a composite score of 31. Both participants were not partaking in regular exercise but did indicate the intention to in the future, with participant 1 planning to take up exercise in the next 6 months, and participant 2 in the next 30 days. Participant 1 had previously been very active, attending highly intensive bootcamp style exercise classes, but since starting a family has focused more on family life had put on weight and avoided exercise entirely. Participant 2 had previously played competitive sports and maintained a high level of physical fitness. In the subsequent years since an injury to his knee he had avoided any physically demanding exercise entirely.

### Intervention

In the current study one-to-one REBT was applied via videoconference. Traditionally REBT studies have used a physical face-to-face environment (see Cunningham and Turner, 2016 for an exception), although research into the delivery of CBTs using distance technology suggests videoconferencing is a viable effective alternative (Day and Schneider, 2002). More recent research into videoconferencing as a viable delivery strategy is positive. Norwood et al. (2018) found the majority of videoconferencing interventions harnessed a positive therapeutic alliance with no significant difference in targeted outcome, while Wang et al. (2019) found small-moderate significant positive differences in distance delivery for musculoskeletal rehabilitation over conventional face to face delivery. However, there are of course limitations to applying psychotherapy via videoconferencing. With no physical interaction it could be argued that the working alliance is compromised because of constrained non-verbal communication through body language, posture, and eye contact (McGinty et al., 2006; Simpson et al., 2021). In addition, technological issues may arise such as loss of connection or computer problems (Connolly et al., 2020), and lack of training in online therapy for practitioners may present a competence barrier (Davies et al., 2020).

The extant REBT literature presents a large range in terms of the number of sessions offered to participants. In a recent large review of the application of REBT, King et al. (2024) reported between 1 and 70 sessions, and 15-min to 140 h contact time. A previous, but still recent, review (Jordana et al., 2023) indicates 2–11 sessions, which is dictated by age. Younger participants require more sessions. In the extant research that uses a single-case design, some papers use as little as one single session (Bowman and Turner, 2022), and 3 sessions (Turner and Barker, 2013). In exercise settings, where evidence is limited but supportive of REBT, Knapp et al. (2023) used 6 sessions, Outar et al. (2018) uses 6 sessions, and Outar et al. (2018) used 5 sessions. Therefore, in line with the prevailing literature, in the current study the REBT intervention consisted of a 5-week REBT program comprising of five 45–60-min one-to-one sessions with self-study homework tasks between the sessions. The sessions were conducted by a 45-year-old male with a primary practicum certificate in REBT. The individual sessions followed a pre-determined structure to ensure procedural synchronicity and reliability across participants (e.g., Outar et al., 2018). The session plan was designed in accordance with guidelines offered by key REBT literature (e.g., Dryden and Branch, 2008; Turner and Bennett, 2017). Following Outar et al.'s (2018) work, the intervention consisted of three phases: psychoeducation, disputation, and reinforcement. It is beyond the scope of the current paper to fully portray the work undertaken with each participant and its inherent idiosyncrasies. However, detailed guidelines for the use of REBT is available in the literature (DiGiuseppe et al., 2014; Ellis and Dryden, 2007; Turner, 2022). But we offer a brief outline of the intervention content that comprised the three phases.

#### Psychoeducation

The intervention was delivered using videoconferencing software Zoom, which allowed for Power Point presentations, whiteboard illustrations, and structured discussions. Across two sessions, both participants where taught the ABC aspects of REBT, and in particular how our beliefs (B) underpin emotional, cognitive, and behavioral consequences (C) to adversity (A). Further, participants learned about the differences between healthy vs. unhealthy negative emotions, and between rational and irrational beliefs. Psychoeducation was managed discursively with discussions taking place between participant and practitioner, although some didactic information delivery was necessary. Participants were asked to provide a brief synopsis of the content at the end of each session to check understanding. After each session participants completed an ABC self-help form as a cognitive task to reflect upon the educational content.

#### Disputation phase

Participants entered this phase with an irrational belief they had formulated in the psychoeducation phase. Across two sessions, participants learned how to dispute this irrational belief, and irrational beliefs *per se*, using three core arguments most commonly written about in REBT literature (Dryden and Branch, 2008; Turner, 2022). In REBT, rather than challenging the adversity (A), we teach clients to challenge irrational belief (B) about A using evidence, logic, and pragmatic disputes. To aid the disputation phase several cognitive disputation tasks were used such as Big I Little i (Lazarus, 1977), friendship disputation, role reversal (Kassinove and DiGiuseppe, 1975; Lipsky et al., 1980), rational emotive imagery (REI; Maultsby, 1971), and Socratic questioning (DiGiuseppe et al., 2014; Turner, 2022). Participants developed alternate rational beliefs to negate their irrational beliefs and disputed these new rational beliefs to ensure they met the criteria of evidence, logic, and pragmatics. As a cognitive homework task participants completed disputation dairies that articulated their attempts to dispute their irrational beliefs, moving through the three disputes independently.

#### Reinforcement phase

During this final session participants first reviewed the main concepts of REBT, recapping on the ABC aspects of REBT, and disputation. Participants reviewed their ABCs which were documented by the practitioner (Tables 1, 2). Any adjustments needed were made collaboratively so that participants left the intervention with an accurate portrayal of their progress through REBT. Then participants planned how they would continue to work on their disputation skills, and how they would maintain their rational beliefs in the longer-term.

**Table 1:** Participant 1's ABC form.

**Activating event (A)**	
**Situation: thinking about exercising**	
**Adversity (inference): I will not be able to do things as I used too**	
**Irrational Belief (iB)**	**Rational Belief (rB)**
Demand: I “Have To” do everything to a high standard and achieve positive judgment	Preference: I would like to do everything to a high standard and receive positive judgment
Depreciation: I am a failure and lazy	Acceptance: I am a fallible complex human and I am not a failure or lazy
Unhelpful consequences (C)	Helpful consequences (C)
Emotional consequence: anxiety, shame, depression	Emotional consequence: embarrassment rather than shame, sadness instead of depression, concerned rather than anxious
Behavioral Consequence: avoidance, Procrastination, Withdrawal	Behavioral Consequence: approach and deal with adversities constructively.
Cognitive consequence: self-downing, thoughts of failure, depreciation	Cognitive consequence: acknowledgment that trying and failing is better than not trying at all. Failure is ok? Feeling worthy? Accept that I am not perfect.

**Table 2:** Participant 2's ABC form.

**Activating event (A)**	
**Situation: thinking about performing physically demanding activity**	
**Adversity (inference): I fear that I will not be able to perform as I would like which would mean I would let myself and others down**	
**Irrational Belief (iB)**	**Rational Belief (rB)**
Demand: I must be successful and always perform as I have in the past	Preference: I would like to be successful but sometimes it is not always possible to perform as I once did
Awfulizing: it will be awful	Anti-awfulizing: If I am not successful it will be bad but not awful
Depreciation: I am a failure and unworthy	Acceptance: this does not make me a failure nor unworthy, it simply means I am a fallible human with both good and bad aspects.
Unhelpful consequences (C)	Helpful consequences (C)
Emotional consequence: anxiety	Emotional consequence: concern
Behavioral consequence: procrastination; lethargic; achy; tired; avoidance; withdrawal	Behavioral consequence: energized; excited; readiness to exercise; approach behavior; motivation
Cognitive consequence: worrying; injury specific thoughts; thinking about an excuse not to do the exercise	Cognitive consequence: rational thoughts about injury; decisive; preparatory thinking

### Measures

#### Irrational beliefs

Originally developed for measuring performance specific irrational beliefs in performance settings such as sport and business, the irrational performance beliefs inventory (iPBI; Turner et al., 2018) has been used successfully in exercise samples (e.g., Turner et al., 2022) including in case study work (Outar et al., 2018). The iPBI comprises 28-items that measure the four core irrational beliefs (demands, awfulizing, frustration intolerance, and depreciation) as well as providing a composite score for all four core irrational beliefs. In the present paper we use only the composite score for brevity. Items are scored on a 5-point Likert-scale ranging from 1 (*strongly disagree*) to 5 (*strongly agree*). The iPBI has good construct (alpha reliability between 0.90 and 0.96), concurrent (medium to large correlations), and predictive (small to medium correlations) validity (Turner et al., 2018).

#### Self-determined motivation

Self-determined motivation was measured using the Behavioral Regulation in Exercise Questionnaire 3 (BREQ3; Markland and Tobin, 2004). The BREQ-3 measures amotivation, external, integrated, introjected, identified, and intrinsic regulation based on the OIT constructs (Ryan and Deci, 2008). The BREQ-3 has shown good factorial validity (Markland and Tobin, 2004; Wilson et al., 2006) and has been extensively used in motivation research. The questionnaire comprises 24 items scored on a five-point Likert scale ranging from 1 (*not true for me*) to 5 (*very true for me*). We adopt the Relative Autonomy Index (RAI; Grolnick and Ryan, 1989; Ryan and Connell, 1989) whereby each regulation subscale is calculated before being weighted and combined with other regulations according to their position on the SDT-continuum (Howard et al., 2020). The RAI is a single score that represents degree of relative autonomy with greater scores indicating more self-determined motivation regulation and lower scores indicating more less self-determined motivation (Markland and Tobin, 2004).

The RAI is not without its critics, with concerns about its statistical validity (Chemolli and Gagné, 2014) and incongruence with the multidimensional nature of motivation regulation (Howard et al., 2020). However, in the current study we are not concerned about using specific regulation types to predict outcomes differentially, rather, we wanted to understand the effects of REBT on relative autonomy as a brief but clear indicator of motivation regulation. The RAI has been used in similar research examining the effects of REBT using single-case procedures to measure changes in motivation regulation as captured in the OIT (e.g., Chrysidis et al., 2020; Davis and Turner, 2020; Turner and Davis, 2019).

#### Readiness, confidence, and motivation to change

In addition to the iPBI and BREQ-3, a three-question exercise specific readiness for change questionnaire was created by the authors to provide evidence of exercise specific changes in cognitive behavior. Administered during the intervention and post-intervention phases (not at baseline) at the same time as the iPBI and RAI, participants were asked to rate on a 1–10 scale between 1 (*not at all*) and 10 (*totally*), “how ready are you to change your exercise behaviors and commit to regular exercise” (readiness), “how confident are you about making these changes” (confidence), and “how motivated are you to change your exercise behaviors and commit to regular exercise” (motivation). Higher scores reflected greater readiness, confidence, and motivation with regards to positive changes in exercise behavior.

#### Regular exercise

Participants were asked to indicate the extent to which they were engaging in regular (3–5 times per week for 20–60 min per session) exercise (i.e., *planned* activity to increase physical fitness such as brisk walking, aerobics, jogging, bicycling, swimming, rowing, etc.) throughout the intervention and post-intervention period (not at baseline) at the same time as the iPBI and RAI. The URICA Exercise Stages of change Questionnaire (Lerdal et al., 2009) was used to frame these questions. Options were: (1) yes I have been for more than 6 months, (2) yes I have been for <6 months, (3) no but I intend to in the next 30 days, (4) no but I intend to in the next 6 months, and (5) no and I do NOT intend to in the next 6 months. Thus, a lower score indicates greater exercise engagement.

### Social validation

A social validation interview was conducted 2 weeks after the post-intervention data had been collected using video conferencing software. The use of social validation in SCEDs is common practice and allows a socially considered appraisal of an intervention's relevancy and efficacy while further supporting the statistical analysis (Page and Thelwell, 2013). The social validation sessions took the format of a semi-structured interview using concise open questioning focusing on the three recommended validation topics as suggested by Page and Thelwell (2013); social significance, social appropriateness, and social importance. Further, as recommended by Page and Thelwell a thematic analysis of the transcribed interviews was conducted to further validate the effectiveness and meaningfulness of the research findings. Using an inductive/deductive approach and incorporating the six phases of thematic analysis as suggested by Braun and Clarke (2006), the current analysis sought to validate and explore the significance, appropriateness, and importance of the findings.

### Data analyses

For analyses of data collected as part of a SCED, visual analyses of each participant's graphed data for target variables (irrational beliefs and self-determined motivation) allows for meaningful interpretation of any significant effects on dependent variables (Barker et al., 2011) so long as instructive guidelines are adhered too. In line with extant REBT research that has adopted a similar SCED (e.g., Outar et al., 2018; Turner and Barker, 2013), we adopted the guidelines from Hrycaiko and Martin (1996). Specifically, if a meaningful change in the dependent variable has occurred, it can be seen graphically based on (a) the number of overlapping data-points between the pre-intervention and post-intervention phases (less is better), (b) the immediacy of an effect following the intervention (sooner is better), and (c) the size of the effect following the intervention (larger is better). Number of overlapping data points is expressed as a percentage (0–100%), immediacy of effect is indicated by change in data in the hypothesized direction at the first intervention phase data point, and effect size was determined using Cohens *d*, which was calculated for baseline phase to intervention phase, intervention phase to post-intervention phase, and from baseline phase to post-intervention phase. Contrary to Cohen's interpretation of effect size in group data (Cohen, 1988). Parker and Vannest (2009) suggest for SCEDs the following effect size interpretations are appropriate: small effect size <0.87; medium effect size 0.87–2.67; and large effect size >2.67.

In addition to visual analyses, statistical analyses were also conducted to examine the phase-to-phase changes in data. As a typical for SCED data (Outar et al., 2018), we first assessed each participant's data for serial dependency via autocorrelation analysis to ensure that the data qualified for parametric tests (Ottenbacher, 1986). Irrational beliefs and RAI scores for both participants yielded non-significant autocorrelation (*r* < 0.63) therefore meeting the requirements for statistical analysis. Therefore, we were able to perform independent sample *t*-tests using SPSS software with statistical significance set at *p* < 0.05 (e.g., Turner and Barker, 2013) for irrational beliefs and RAI for baseline to intervention phases, and for intervention to post-intervention phases (four *t*-tests in total). We also conducted Tau-*U* tests following guidelines by Parker et al. (2011) using their online Tau-U calculator (http://www.singlecaseresearch.org/calculators/tau-u). The Tau-*U* test was designed for single-case research and reflects the proportion of data that are different (or non-overlapping) in the intervention phase in comparison with the baseline phase (e.g., Christakou et al., 2019). Tau-U indicates the percentage of data that changes across phases and accounts for baseline trend. In the case of the present paper, these phases are baseline-intervention, intervention-post-intervention, and baseline-post-intervention. Chen et al. (2016) suggest a Tau-U value of 0.47 and above indicates a large effect, whilst Rakap (2015) suggests that 0.66 and above indicates that the intervention is effective and 0.93 and above indicates that the intervention is very effective. Thus, in the current paper we use cut-offs of <0.47, 0.47–66, ≥0.93 to be indicators of small, moderate, and large effects.

### Participant engagement

#### Participant 1

It was evident that participant 1 actively engaged with the education stage of the intervention regularly asking questions, relating the principles being taught to her own situation and experiences. At times she struggled to articulate the principles of REBT when asked for her understanding, but by the end of the psychoeducation phase she was able to grasp the ABC of REBT. In order to ensure she understood the REBT principles fully, the psychoeducation phase extended slightly into the disputation phase, therefore for participant 1 the psychoeducation phase lasted 2.5 sessions. Participant 1 invested in the work well, at times becoming emotional in the sessions when exploring her irrational beliefs, and later in the sessions started to evidence promising change intensions. In the disputation phase, the participant had some difficulty moving away from her rigid thinking, and repeated disputation was undertaken until the participant was able to challenge her beliefs successfully.

During the friendship disputation task participant 1 became tearful especially when reflecting on why she had flexible beliefs for her children yet held extreme and rigid beliefs for herself. This was a powerful realization for her and a pivotal point in the intervention. In the final session participant 1 recounted an incident in which she used her new rational belief as part of a coping statement stored in her mobile phone. Reportedly, she wanted to go for a jog and had the thought that others would look down on her (A) which would be awful and prove she was worthless (B). She reported that she realized that even if this event (A) did occur, it would not be awful and would not mean that she was worthless—her worth does not depend on the views of others. This occurrence was another landmark moment in the intervention because it illustrated her ability to independently apply REBT in her life. In addition, she was also able to keep her unhealthy emotions at bay when entering an exercise class by using REI prior to attending the class, and by repeating her rational belief to herself as she passed through the entrance doorway to the class. Of note, during the disputation sessions there were several instances of change talk. For instance, the participant asked the practitioner's advice on purchasing new trainers for exercise.

#### Participant 2

It was evident that participant 2 fully engaged in the education phase and appeared very relaxed, and was very matter of fact within the disputation phase in particular. He grasped the concepts quickly and understood how they related to his own situation. Finding the main irrational belief to work on was somewhat challenging with participant 2, but after some in-depth exploration we were able to frame an irrational belief that spoke to the participant deeply enough to stimulate work toward its amelioration. Participant 2 struggled to fully accept his newly developed rational belief, and whilst he intellectually understood and agreed with his new belief, he had to work hard to connect with the belief more deeply and seemed stuck in his irrationality. At the end of the work participant 2 had a plan to reinforce the rational belief knowing that his challenge was to work on strengthen his connection to the new rational belief.

Throughout the intervention, participant 2 worked to internalize the REBT concepts by partaking thoroughly in all the session and in between session exercises that were set. He particularly embraced REI and Role Reversal. In session four he added his rational belief to his mobile phone and reported regularly reading it during the day. Importantly, as the disputation phase progressed participant 2 reported an increase in regular exercise participation and started to use more and more change talk which focused on preparing for exercise. For instance, he spoke about ensuring his bike was fit for purpose, checking the tire, and making sure his gear was ready, and talked about going for longer walks and exploring the local countryside.

## Results

### Irrational beliefs

#### Participant 1

**Figure 1:**
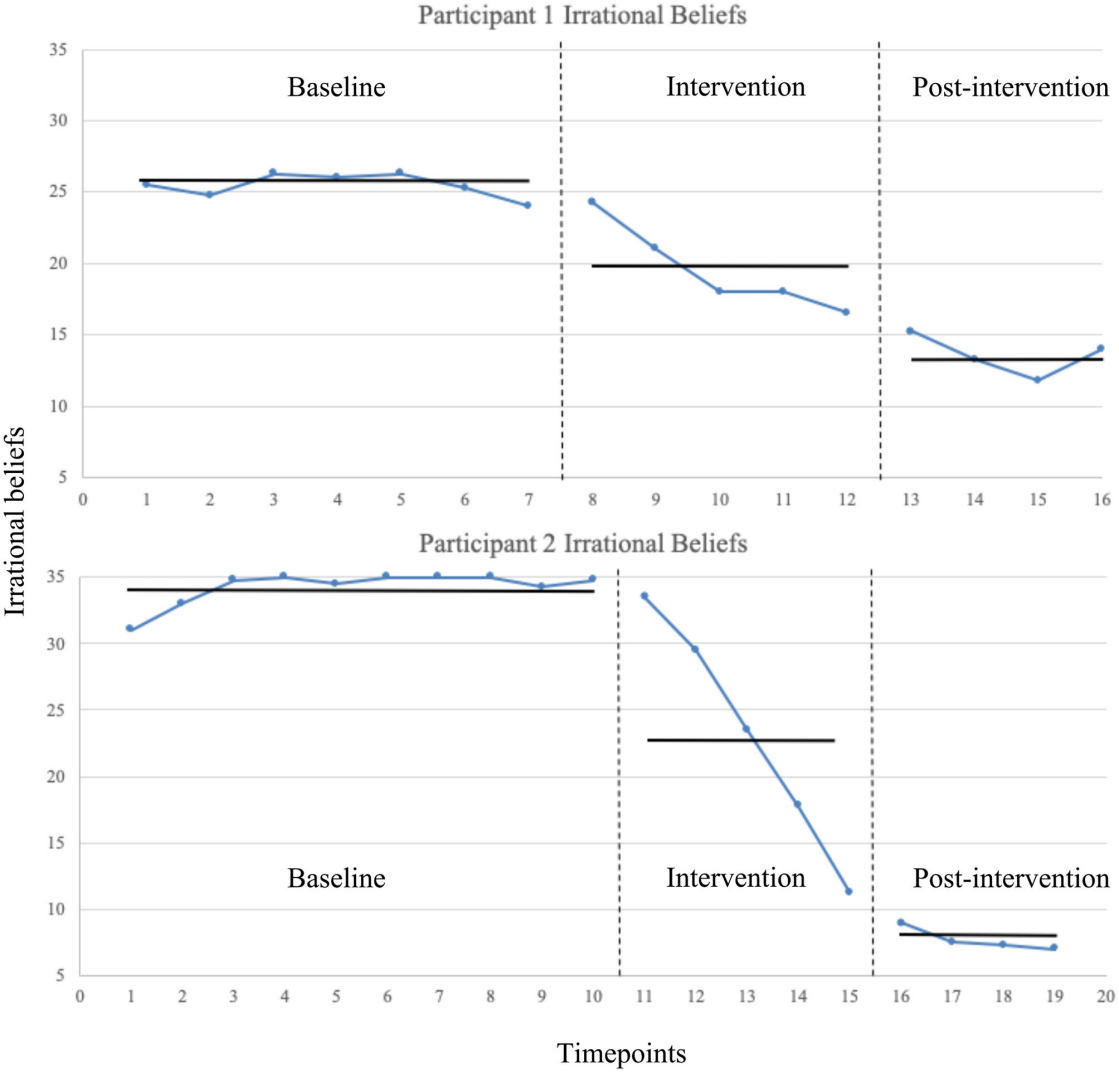
Irrational beliefs measured using the iPBI at each of the timepoints across baseline, intervention, and post-intervention phases. Vertical dotted lines indicate phase transition. Means levels at each phase shown with horizontal solid lines.

Irrational beliefs decreased from baseline to intervention immediately after the second session with no overlapping data points. A decrease was observed from intervention to post-intervention and continued to decrease until the last data point which increased but did not overlap with any intervention phase scores. The statistical analysis for Mean levels at each phase revealed a significant and large reduction from baseline (M = 25.24, SD = 0.84) to intervention phase [(M = 19.55, SD = 3.09), *t*_(10)_ = 4.87, *p* < 0.001, *d* = 2.60)], and a significant and medium-large reduction from intervention to post-intervention phase (M = 13.56, SD = 1.46), *t*_(7)_ = 3.53, *p* = 0.010, *d* = 2.47. From baseline to post-intervention there was a significant and large reduction, *t*_(9)_ = 17.42, *p* < 0.001, *d* = 9.97. For the Tau-*U* analyses, baseline trend correction was not required (Tau = −0.19, SDtau = 0.32, Z = −0.60, *p* = 0.55). There was a large reduction from baseline to intervention phase (Tau = −0.94, SDtau = 0.35, Z = −2.68, *p* =0.007), from intervention phase to post-intervention phase (Tau = −0.10, SDtau = 0.41, Z = −2.45, *p* = 0.014), and from baseline to post-intervention phase (Tau = −0.10, SDtau = 0.38, Z = −2.65, *p* =0.008). See Figure 1 for graphed data.

#### Participant 2

Irrational beliefs decreased from baseline to intervention immediately after the first session with no overlapping data points. A decrease was observed from intervention to post-intervention with no overlapping data points, but then a plateau was observed due to a floor effect. The statistical analysis for Mean levels at each phase revealed a significant and medium reduction from baseline (M = 34.23, SD = 1.29) to intervention phase [(M = 23.10, SD = 8.92), *t*_(13)_ = 4.01, *p* < 0.001, *d* = 1.75], and a significant and medium-large reduction from intervention to post-intervention phase (M = 7.69, SD = 0.90), *t*_(7)_ = 3.39, *p* = 0.012, *d* = 2.43. From baseline to post-intervention there was a significant and large reduction, *t*_(12)_ = 37.30, *p* < 0.001, *d* = 23.86. For the Tau-*U* analyses, baseline trend correction was not required (Tau = 0.31, SDtau = 0.25, Z = 1.25, *p* = 0.21). There was a moderate-large reduction from baseline to intervention phase (Tau = −0.92, SDtau = 0.33, Z = −2.82, *p* = 0.005), a large reduction from intervention phase to post-intervention phase (Tau = −0.10, SDtau =0.41, Z = −2.45, *p* = 0.014), and from baseline to post-intervention phase (Tau = −0.10, SDtau = 0.35, Z = −2.83, *p* = 0.005). See Figure 1 for graphed data.

### RAI

#### Participant 1

**Figure 2:**
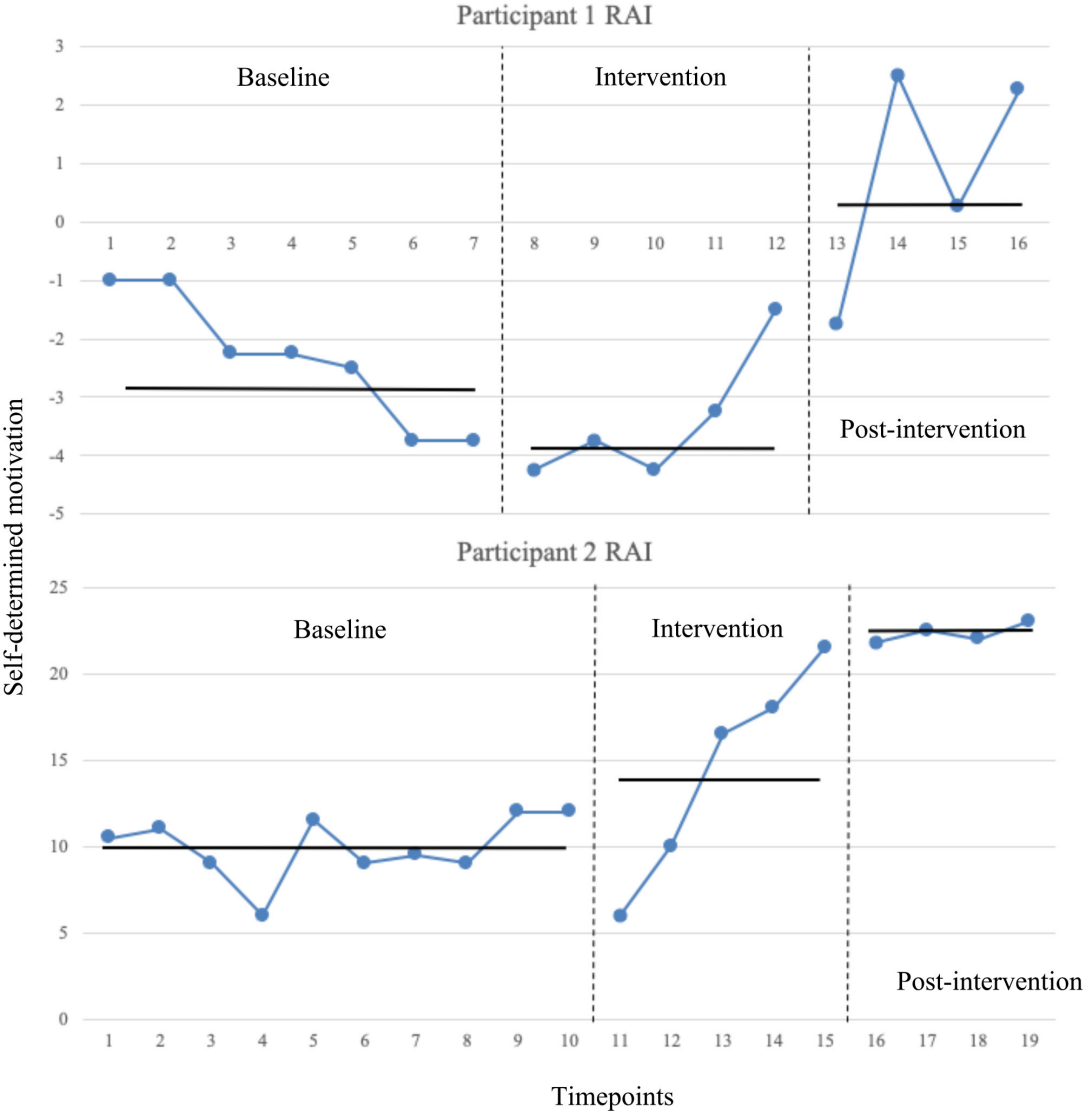
RAI (self-determined motivation) data across timepoints for both participants. RAI measured using the BREQ-3 at each of the timepoints across baseline, intervention, and post-intervention phases. Vertical dotted lines indicate phase transition. Means levels at each phase shown with horizontal solid lines.

RAI decreased from baseline at the onset of the intervention with all intervention data points overlapping with baseline data points. After sessions 5, RAI increased to above the baseline Mean levels, and at post-intervention RAI increased again with only one overlapping data point. The statistical analysis for Mean levels at each phase revealed a non-significant and medium reduction from baseline (M = −2.36, SD = 1.13) to intervention phase [(M = −3.40, SD = 0.07), *t*_(10)_ = 1.57, *p* = 0.147, *d* = 1.30], and a significant and large increase from intervention to post-intervention phase (M = 0.81, SD = 0.08), *t*_(7)_ = 4.03, *p* = 0.005, *d* = 56.01. From baseline to post-intervention there was a significant and large increase, *t*_(9)_ = 3.44, *p* = 0.007, *d* = 3.96. For the Tau-*U* analyses, baseline trend correction was required (Tau = −0.86, SDtau = 0.32, Z = −2.70, *p* = 0.007). There was no change from baseline to intervention phase (Tau = −0.02, SDtau = 0.35, Z = −0.08, *p* = 0.935). There was a moderate-large increase from intervention phase to post-intervention phase (Tau = 0.90, SDtau = 0.41, Z = 2.21, *p* = 0.028), and from baseline to post-intervention phase (Tau = 1.50, SDtau = 0.38, Z = 3.97, *p* < 0.001). See Figure 2 for graphed data.

#### Participant 2

RAI decreased from baseline at the onset of the intervention, but there was a steady increase within the intervention phase with two of the five data points overlapping with baseline data points. At post-intervention RAI increased again slightly and remained elevated above baseline and intervention levels with no overlapping data points. The statistical analysis for Mean levels at each phase revealed a non-significant and small increase from baseline (M = 9.95, SD = 1.85) to intervention phase [(M = 14.40, SD = 6.28), *t*_(13)_ = 2.13, *p* = 0.052, *d* = 0.96], and a significant and medium increase from intervention to post-intervention phase (M = 22.31, SD = 0.55), *t*_(7)_ = 2.48, *p* = 0.042, *d* = 1.73. From baseline to post-intervention there was a significant and large increase, *t*_(12)_ = 12.87, *p* < 0.001, *d* = 8.92. For the Tau-*U* analyses, baseline trend correction was not required (Tau = 0.24, SDtau = 0.25, Z = 0.98, *p* = 0.33). There was no change from baseline to intervention phase (Tau = 0.42, SDtau = 0.33, Z = 1.29, *p* =0.198). There was a large increase from intervention phase to post-intervention phase (Tau = 1.00, SDtau = 0.41, Z = 2.45, *p* = 0.014), and from baseline to post-intervention phase (Tau = 1.00, SDtau = 0.35, Z = 2.83, *p* = 0.005). See Figure 2 for graphed data.

### Readiness, motivation, and confidence to change

#### Participant 1

**Figure 3:**
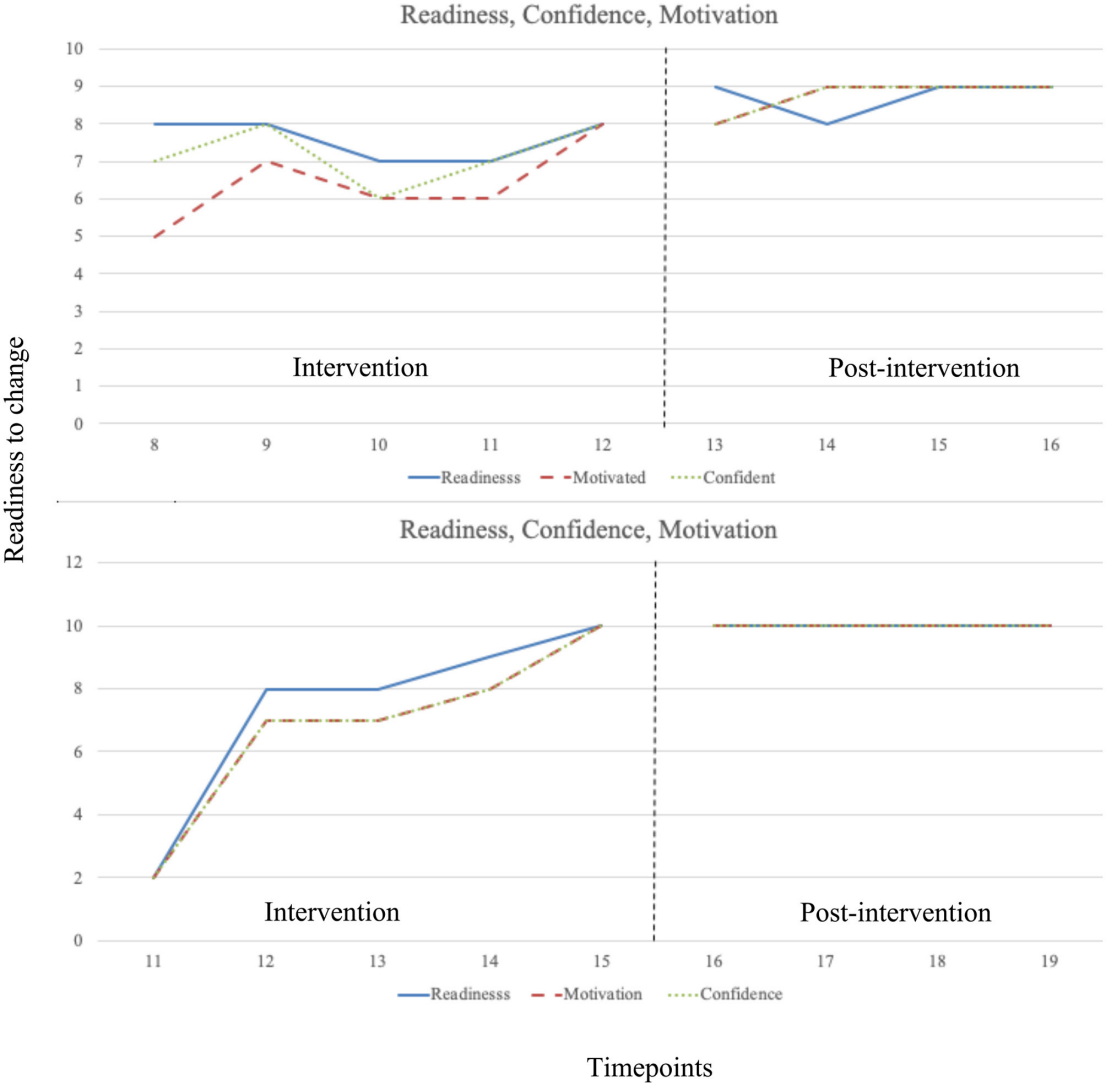
Intervention and post-intervention phase readiness, confidence, and motivation data at each timepoint across baseline, intervention, and post-intervention phases. Vertical dotted lines indicate phase transition.

Scores increased steadily throughout the intervention phase and maintained at their highest in the post-intervention phase with no overlapping data points. There was a significant medium-large increase from the intervention phase to the post-intervention phase in readiness to change, Mincrease = 1.15, *t*_(7)_ = 3.25, *p* = 0.014, *d* = 2.19, motivation to change, Mincrease = 2.35, *t*_(7)_ = 3.25, *p* = 0.014, *d* = 2.67, and confidence to change, Mincrease = 1.55, *t*_(7)_ = 3.80, *p* = 0.007, *d* = 2.24. See Figure 3 for graphed data. For the Tau-*U* analyses, there was a moderate-large increase from baseline to intervention phase in readiness to change (Tau = 0.85, SDtau = 0.41, Z = 2.08, *p* = 0.037), large increase motivation to change (Tau = 0.95, SDtau = 0.41, Z = 2.33, *p* = 0.020), moderate-large increase motivation to change (Tau = 0.90, SDtau = 0.41, Z = 2.20, *p* = 0.028).

#### Participant 2

Scores increased markedly across the first two REBT sessions in the intervention phase, continuing to increase steadily throughout the intervention phase. Scores were highest at the end of the intervention phase, maintaining throughout the post-intervention phase with no overlapping data points. There was a non-significant medium increase from the intervention phase to the post-intervention phase in readiness to change, Mincrease = 2.60, *t*_(7)_ = 1.64, *p* = 0.145, *d* = 1.17, motivation to change, Mincrease = 3.20, *t*_(7)_ = 2.14, *p* = 0.070, *d* = 1.53, and confidence to change, Mincrease = 3.20, *t*_(7)_ = 2.14, *p* = 0.070, *d* = 1.53. See Figure 3 for graphed data. For the Tau-*U* analyses, raw scores were identical for all three variables at each datapoint, and there was a moderate-large increase from baseline to intervention phase in all three variables (Tau = 0.80, SDtau = 0.41, Z = 1.96, *p* = 0.050).

### Regular exercise

#### Participant 1

Regular exercise increased from intervention to post-intervention immediately after the intervention phase has ceased, with no overlapping data points. There was a significant increase from the intervention phase (M = 3.00) to the post-intervention phase (M = 2.00) in regular exercise, Mdecrease = 1.00, *t*_(7)_ = 316.95, *p* < 0.001. For the Tau-*U* analyses, there was a large increase from the intervention to post-intervention (Tau = 1.00, SDtau = 0.41, Z = −2.45, *p* = 0.014).

#### Participant 2

Regular exercise increased from intervention to post-intervention, and this increase occurred after the second REBT session, with no overlapping data points. There was a non-significant increase from the intervention phase (M = 2.40) to the post-intervention phase (M = 2.00) in regular exercise, Mdecrease = 0.40, *t*_(7)_ = 1.50, *p* = 0.176. For the Tau-U analyses, there was a small (negligible) increase from the intervention to post-intervention (Tau = 0.40, SDtau = 0.41, Z = −0.98, *p* = 0.327).

### Social validation

Social validation interviews and thematic analysis indicated both participants experienced positive changes to exercise behaviors and cognitions, which were maintained post-intervention. They also reported changes in other aspects of their lives such as the workplace. Overall, social validation data indicated that the intervention was socially significant, appropriate, and important to participants (Page and Thelwell, 2013).

A common theme for both participants was “positive cognitive and behavioral change.” Participant 1 reported that the intervention changed how she thought about exercise stating “it's not a chore…or a burden that I have to do, it's more a thing that kind of fits in and a thing I want to do” and “I can change how I feel…, I don't have to be anxious and the negative feelings aren't caused by the situation it's the stuff that I'm feeling.” Participant 2 reported an increase in exercise behavior stating “certainly exercising a lot more over the last 6 weeks of the intervention than I have been previously” and a change from black and white thinking to more logical thinking “before, earlier it was really black and white and I have extreme viewpoints, I kind of changed as I went through and it kind of made me think more logically.”

The efficacy and delivery of the intervention was confirmed by both participants under a common theme of “credible intervention delivery.” In reference to the delivery, participant 2 stated “it was really clear every time you explained what we were doing …it was very logical and well methodically planned.” Participant 1 commenting on the benefits of videoconferencing over face-to-face delivery stating, “I feel more comfortable talking non-face to face, so I found it easier.” Although the delivery via videoconferencing worked well for participant 1 she did reflect on the inability of the researcher to see non-verbal gestures “maybe the words and the gesticulating with my hands waving around things like that but no I think for me personally I would say I feel more comfortable talking non face to face.”

An important aspect of the intervention was educating and facilitating a change from depreciation to unconditional self-acceptance. Acknowledging the interventions effectiveness, the thematic analysis found self-acceptance as a main theme for participant 1. For example, participant 1 stated “working on not worrying so much about…having to be perfect and not worrying about what other people are thinking, no one is perfect” and “I see it as more normal, not that it wasn't normal before, I don't know, I feel like I am more in with the crowd now” illustrates how participant 1 internalized her new rational thinking and related it to others while normalizing exercise as part of her new identity.

## Discussion

In the current study we aimed to examine to extent to which applying REBT with non-exercise engaged participants could facilitate a positive change in exercise behavior. Based on previous work in the field (e.g., Davis and Turner, 2020; Knapp et al., 2023; O'Connor, 2018; Outar et al., 2018) it was hypothesized that REBT would facilitate a reduction in irrational beliefs and an increase in self-determined motivation, and subsequent increases in readiness, confidence, and motivation to engage in exercise. There is scant research into the effects of REBT in exercise settings, and therefore this paper makes a novel contribution to research by testing the extent to an REBT intervention could affect exercise-related outcomes. The results indicate that REBT brought about a reduction in irrational beliefs and an increase in self-determined motivation. Participants also reported subsequent increases in readiness, confidence, and motivation to change, and that these changes in how they think about exercise encouraged them to exercise more. Thus, the findings are promising.

Ellis (1994), writing about exercise engagement, offered an example of a rational approach to exercise:

It's most desirable that I exercise regularly, but there's no law of the universe that says that I have to do so. It's not awful that I'm behaving so foolishly, and my stupid acts never make me a stupid, rotten person. Though I've failed many times before to exercise, I am not a total failure. I can now mend my ways, work on my intolerance for frustration and force myself to exercise if it's the last thing I do. For if I don't exercise, that may well be the last thing I don't do (p. 253).

Ellis' (1994) example of a rational approach to exercise above does capture somewhat where the participants in the current study ended up at the end of their REBT work. However, there is one aspect of Ellis' statement that diverges from the work completed in the current paper. Rather than using REBT to encourage a participant “force myself to exercise” (Ellis, 1994, p. 253), based on the data presented in the present paper, it seems that REBT can encourage more self-determined behavioral regulation in relation to exercise. The idea of forcing oneself to undertake a behavior sits more comfortably alongside irrational beliefs, rather than rational beliefs, in REBT. To illustrate, the dogmatic demand to perform exercise behavior (“I must exercise”) and the idea that my self-worth is dependent upon my exercise behavior (“if I do not exercise, I am a complete loser”), reflects introjected regulation in the OIT, characterized by self-pressure and contingent self-worth (Lonsdale and Hodge, 2011), whereby the motivation for the behavior has been partially, but not fully, internalized (Hurst et al., 2017). Introjected regulation has been found to be associated with greater physical activity levels (Gillison et al., 2009), but on the whole, evidence indicates that found that more self-determined motivation regulation predicts positive exercise behavior (Teixeira et al., 2012). As such, rather than helping sedentary adults to force themselves to exercise, it is perhaps more sensible and effective in the longer-term to help them to move toward more intrinsic motives for partaking in exercise.

When Albert Ellis wrote in 1994 that irrational beliefs combined with a displeasure about exercising, block people from exercising, there was little externally valid data to support his clinical anecdotal reports. We aimed to meet a research need in the REBT and exercise literature, by examining the effects of REBT on self-determined exercise motives, and subsequent readiness, confidence, and motivation to engage in exercise. We also conducted social validation interviews post-REBT to understand the idiographic effects of REBT on participants' approach to and engagement in exercise. Our approach offers an idiographic perspective on REBT applied with sedentary adults, adopting a rigorous single-case experimental design (adhering to Hrycaiko and Martin, 1996), allowing data triangulation and visual and statistical analyses.

The results from the visual and statistical analysis indicate that the REBT intervention was effective at reducing irrational beliefs for both participants and this effect was jointly observed immediately after the second REBT session. This may have been because the first session was an introductory session intended to gain insight into the participants exercise behavior, rather than apply REBT for the reduction of irrational beliefs. This finding echoes previous research whereby changes in irrational beliefs do not necessarily occur immediately after the first session (Cunningham and Turner, 2016; Turner and Davis, 2019; Turner et al., 2020b). Ultimately, it is the movement toward disputing irrational beliefs that should drive irrational beliefs reduction, and this does not normally take place in the first session, unless the work comprises a single session (e.g., Bowman and Turner, 2022; Turner et al., 2014).

The idiographic single-case approach we took to the paper offers some important advantages over grouped data when trying to understand intervention effects. For example, in the present data it can be seen that whilst participant 2's RAI scores show a steady increase as the intervention progresses, the pattern for participant 1's data suggest something different. For participant 1, during REBT, RAI actually remains low until after session 4, after which point RAI scores start to climb. Also, whilst graphed data clearly show that participant 2 reports increases in change readiness, confidence, and motivation, and in regular exercise from the intervention phase to the post-intervention phase, just as participant 1 reports, the statistical analyses render these changes statistically non-significant. For the statistical analyses data were averaged for each phase, thus losing the high-resolution change data afforded by the single-case approach. The idiographic single-case design we adopted in the present paper allows us to detect these idiosyncratic patterns of change for each participant, speaking to the advantages of this design over group-based deigns where data are amalgamated across participants. Rather than differential change rates being a bug of the design, it is a feature, allowing researchers to understand that each participant is unique. Whilst REBT appears to stimulate positive changes in a range or markers (see King et al., 2023; for a review), rates of change will differ between participants based on a range of factors outlined by Turner (2022) such as metacognitive ability (Weil et al., 2013), willingness to introspect, commitment to the REBT process, resistance to conversation of a psychotherapeutic nature, personality and strength of working alliance (e.g., Menon et al., 2015).

Ultimately, not every client with respond to REBT in the same manner, a fact that is echoed in most previous literature that has used idiographic methods (see Bowman and Turner, 2022, for a good example of this). It is important that we tailor REBT to clients and not assume that REBT will work in the same way and at the same pace for everybody (Dryden and Neenan, 2015). Individual differences in data patterning are of course in part dependent on the individual differences that participants bring to the intervention process. For example, participant 1 was a middle-aged female with a young family, a working husband, and a new house. Participant 2 was a male in his early thirties, unmarried, with no children. It is unlikely that these two very different people will react in the same way to REBT, although, there is some clear corroboration between the two participants' data patterns (i.e., reduced IBs and increased RAI).

The current study also offers some support for a sparsely reported mode of REBT delivery, that of videoconferencing, or VREBT. Cunningham and Turner (2016) offered some evidence for the use of VREBT in their work with Mixed Martial Arts athletes, and the present study offers further support for this mode. One participant in the current study actually preferred videoconferencing to physical delivery, but caveated this with the fact that non-verbal gestures were absent from the interaction. Videoconference-delivered CBT (VCBT) enables remote working, which certainly fits zeitgeist of the modern post-COVID-19 working world (Fernández-Álvarez and Fernández-Álvarez, 2021), and has the benefit of real-time face-to-face communication (Bergman et al., 2003; Matsumoto et al., 2021; Rees and Stone, 2005). Previous research suggests that the benefits of using a video conferencing delivery outweigh the lack of physical contact time (Wang et al., 2019; Norwood et al., 2018), but the physical separation between client and practitioner may create limitations such as time lags and poor eye contact that could interfere with the interaction (Matsumoto et al., 2021). In addition, network connectivity issues can stall sessions, or indeed could actually end the session abruptly if the connection issues are serious. But in the clinical space, rigorous clinical trials (RCTs) indicate that VCBT is not inferior to face-to-face CBT in treating depression and posttraumatic stress disorder (e.g., Acierno et al., 2016; Egede et al., 2015), as seems especially effective for depressive symptoms (Matsumoto et al., 2021). For REBT specifically, Stefan and David (2013) compared physical REBT with VREBT and found that both modes equally reduced distress and irrational beliefs, and that the mode did not significantly impact upon the perceived quality of the working alliance. However, research does point to the idea that physical therapy is more effective in helping foster working alliance (Norwood et al., 2018). More recently Mercadal Rotger and Cabré (2022) compared the establishment of the therapeutic alliance (TA) between physical and online therapy, finding that the physical mode was superior. The full extent to which VREBT is more or as effective face-to-face REBT is still to be determined, but is a viable and needed area of future research as more sport and exercise psychologists adopt remote working modes of service provision.

### Study limitations

Although we applied a robust single-case experimental design, researchers may wish for a randomized control trial (RCT) to be confident in the generalisability in the findings to warrant application of REBT to exercise behavior (Jordana et al., 2023; see Toth et al., 2023). For example, Nejati et al. (2024) used a small RCT design to test the effects of REBT on performance under pressure, finding significant decrease in irrational beliefs and social anxiety and an improvement in performance under pressure in the experimental group, compared to a placebo control group. The results of the current study communicate the effects of REBT on two adults, and as such, findings cannot be generalized to the population. An RCT might wish to apply REBT with participants who at a screening stage report a sedentary lifestyle alongside high irrational beliefs, compared to a compassion treatment group and waitlist control group.

We could also have been more robust with our measurement of exercise behavior. We used self-reported exercise engagement, but the positive effects of REBT on exercise might not relate to actual exercise participation. We could include self-reported measures such as the 7-day physical activity recall (PAR; Blair et al., 1985) or the Godin leisure-time exercise questionnaire (LTEQ; Godin and Shephard, 1985), alongside accelerometery or pedometery to assess actual movement, and behavioral markers such as exercise attendance, number of exercise relapses, and exercise dropout (e.g., Teixeira et al., 2012). Whilst we did collect data on readiness, confidence, and motivation to engage in exercise, we did not assess these markers at baseline. We were conscious of the Hawthorne effect in this study, whereby individuals may modify their behavior in response to their awareness of being observed (e.g., McCarney et al., 2007). Our concern was that if we monitored exercise intention throughout baseline, that participants would start to engage in more exercise and thus violate a key assumption for visual analysis, that of a stable baseline or a baseline pattern that moves in the opposing direct to hypothesized intervention effects. But this lack of baseline measurement means that we only tracked exercise intention through the intervention and post-intervention phases, which demonstrate an increase and maintenance of this increase.

In addition, ideally we would monitor post-intervention effects over a long period than 8 weeks in order to ascertain to what extent effects were maintained. This is a flaw of much single-case research, and indeed applied research *per se*, owed in part to the realities of conducting research in a timely manner, and not wanting to overburden participants. In essence, we cannot say whether the REBT intervention brought about long-term change, nor so we suggest that effects would be maintained past the research study period. Perhaps the REBT paper with the longest continuous maintenance period is that of Turner and Barker (2013) in which one participant records 10 weeks of post-intervention data, showing that changes in irrational beliefs and anxiety were maintained after a three-session intervention. A rare study by Chrysidis et al. (2020) used a 1-year post-REBT maintenance datapoint, showing that the effects of a five-session REBT intervention were maintained in two participants. Thus, research in the future should extend the measurement of effects beyond a number of weeks, to a number of months, and perhaps years.

Finally, in the real world, outside of the context of a scientific study, a practitioner might use a multi-modal and integrative approach to applying REBT, using a number of cognitive, emotive, and behavioral methods (Ellis, 1994; Turner, 2022). The present study reports REBT as a mostly cognitive approach to REBT where prominence was placed upon belief change using verbal disputation. Future research could apply a broader range of techniques (e.g., exposure; Beck and Haigh, 2014) to maximize the effects of REBT.

### Practical implications

In light of study strengths and limitations, there are some potential useful practical implications of the present study. Whilst we cannot attest to the longer-term effects of REBT in exercise motives and intentions, in the short-term, REBT was useful in bringing about positive change. Thus, practitioners tasked with helping patients or clients to become more physically active might consider brief (five-session) REBT in their work. If applied, there are details in the current paper, owing to its idiographic focus, that could help enhance the effects of REBT. For example, for participant 1 In the disputation phase, there was some difficulty moving away from irrational beliefs rigid thinking, and so there was a need to repeat the disputation aspects of REBT. When working with clients it is important not to rush any of the REBT processes, and repetition of key processes such as disputation is recommended. Similarly, with participant 2 there was difficulty locating the main irrational beliefs at the center of his issues. It is important not to force this part of REBT, because we might arrive at an irrational belief out of convenience rather than accuracy (Turner, 2022). Once participant 2 has accurately articulated their irrational belief, then work could progress forthrightly. But it is the client that dictates whether or not the irrational belief has been located, because it is they who can truly understand its impact once brought to fruition. Clients will also at times show some signs of resistance to the newly developed rational beliefs (Ellis, 2002), as participant 2 demonstrates. This is typical, because clients may have deeply held their irrational belief for many years, perhaps decades depending on the age of the client, so adopting a new belief that counters the irrational beliefs may not be easy. Client and practitioner must work to strengthen the new belief, rehearse, and find ways to utilize the belief in real life. For example, rational self-talk can help clients to practice and imbed their new rational beliefs (e.g., Turner et al., 2020c). To this point, a key moment for participant 2 was when she went jogging and was able to apply her rational belief to the prospect of social disapproval. Only be facing the A can the new B ne applied and tested, so in REBT practitioners should not forget the utility and importance of behavioral homework assignments (DiGiuseppe et al., 2014). REBT can seem very “cognitive” because much time is spent on cognitive restructuring, but REI and behavioral assignments offer emotive and behavioral techniques that can support client change.

## Conclusion

Finding ways to help people to undertake more exercise is an important public health need. One potential method is REBT, which can be applied on a one-to-one basis using remote videoconferencing facilities. In present study, REBT was applied with two sedentary adults, and we examined changes in irrational beliefs, self-determined motivation to exercise, readiness, confidence, and motivation to exercise, and exercise engagement. Using an idiographic single-case experimental design, we found that overall REBT was effective in bringing about positive change. However, there were individual differences in patterns and magnitude of change. The findings support some previous research and theorizing, but also extend the area by providing data to indicate the effects of REBT on exercise related outcomes. Future research needs to build on these findings and investigate the use of REBT to reduce exercise avoidance in different populations, different communities, and different contexts and continue the exploration of novel delivery strategies that can reach as many people as possible.

## Data Availability

The raw data supporting the conclusions of this article will be made available by the authors, without undue reservation.
